# Supplementary material for: Increased consumption of ultra-processed foods and worse diet quality in colorectal cancer patients after colostomy: A prospective study
Source: PLoS One. 2025 Jan 9;20(1):e0310320. doi: 10.1371/journal.pone.0310320 (PMC11717310; doi:10.1371/journal.pone.0310320)
Supplement: S1 Table — (DOCX) [file pone.0310320.s001.docx]

**S1 Table.** Effect size and observation power of the food consumption variables of the Brazilian Healthy Eating Index and processing level (NOVA classification)

| **Variable** | **Time** | **Effect size** | **Observation power** |
| --- | --- | --- | --- |
| **Total grains** | T0-T1 | -0.09 | 0.11 |
|  | T0-T2 | -0.19 | 0.21 |
|  | T1-T2 | -0.14 | 0.12 |
| **Whole grains** | T0-T1 | -0.14 | 0.16 |
|  | T0-T2 | -0.14 | 0.15 |
|  | T1-T2 | -0.45 | 0.46 |
| **Total fruit** | T0-T1 | -0.47 | 0.69 |
|  | T0-T2 | -0.61 | **0.85** |
|  | T1-T2 | -0.45 | 0.29 |
| **Whole fruit** | T0-T1 | -0.40 | 0.57 |
|  | T0-T2 | -0.67 | **0.91** |
|  | T1-T2 | -0.22 | 0.19 |
| **Total vegetables** | T0-T1 | -0.08 | 0.10 |
|  | T0-T2 | -0.39 | 0.53 |
|  | T1-T2 | -0.10 | 0.10 |
| **Dark green and orange vegetables and legumes** | T0-T1 | -0.11 | 0.13 |
|  | T0-T2 | -0.31 | 0.15 |
|  | T1-T2 | -0.38 | 0.13 |
| **Milk and dairy products** | T0-T1 | -0.11 | 0.12 |
|  | T0-T2 | -0.31 | 0.39 |
|  | T1-T2 | -0.38 | 0.37 |
| **Meat, eggs and legumes** | T0-T1 | -0,15 | 0,17 |
|  | T0-T2 | -0,09 | 0,11 |
|  | T1-T2 | 0,00 | 0,05 |
| **Oils** | T0-T1 | -0,09 | 0,11 |
|  | T0-T2 | -0,21 | 0,24 |
|  | T1-T2 | -0,27 | 0,24 |
| **Saturated fat** | T0-T1 | -0,26 | 0,37 |
|  | T0-T2 | -0,13 | 0,14 |
|  | T1-T2 | -0,23 | 0,20 |
| **Sodium** | T0-T1 | 0.10 | 0.12 |
|  | T0-T2 | -0.61 | **0.87** |
|  | T1-T2 | -0.64 | 0.73 |
| **Energy content from SoFAAS** | T0-T1 | -0.28 | 0.35 |
|  | T0-T2 | -0.18 | 0.20 |
|  | T1-T2 | -0.19 | 0.16 |
| **Total BHEI-R** | T0-T1 | -0.44 | 0.65 |
|  | T0-T2 | -0.66 | **0.91** |
|  | T1-T2 | -0.02 | 0.06 |
| **Raw or minimally processed** | T0-T1 | -0.59 | **0.86** |
|  | T0-T2 | -0.36 | 0.49 |
|  | T1-T2 | 0.31 | 0.29 |
| **Culinary ingredients** | T0-T1 | -0.22 | 0.27 |
|  | T0-T2 | -0.18 | 0.20 |
|  | T1-T2 | 0.16 | 0.14 |
| **Processed** | T0-T1 | -0.16 | 0.18 |
|  | T0-T2 | -0.12 | 0.13 |
|  | T1-T2 | -0.16 | 0.14 |
| **Ultra-processed** | T0-T1 | 0.80 | **0.98** |
|  | T0-T2 | 0.45 | 0.65 |
|  | T1-T2 | -0.50 | 0.41 |

Abbreviation: BHEI-R: Brazilian Healthy Eating Index
