# Supplementary material for: Increased consumption of ultra-processed foods and worse diet quality in colorectal cancer patients after colostomy: A prospective study
Source: PLoS One. 2025 Jan 9;20(1):e0310320. doi: 10.1371/journal.pone.0310320 (PMC11717310; doi:10.1371/journal.pone.0310320)
Supplement: S2 Table — (DOCX) [file pone.0310320.s002.docx]

| S2 Table. Differences in the general characteristics of patients who remained in the study and those lost to follow-up | | | |
| --- | --- | --- | --- |
| Variables | **Patients that participate in T0 and T1 and/or T2**  **(n=31)^**^** | **Follow-up losses (n=15)** | ***p-value** |
|  | % (n) | |  |
| Age (years), mean ± SD | 61.9 ± 7.88 | 57.8 ± 18.25 | 0.290 |
| Sex |  |  | 0.171 |
| Male | 54.8 (17) | 33.3 (5) |  |
| Female | 45.2 (14) | 66.7 (10) |  |
| Family income (minimum wages)^* £^ |  |  | 0.110 |
| < 1 | 9.7 (3) | 6.7 (1) |  |
| ≥ 1 - 2 | 35.5 (11) | 6.7 (1) |  |
| ≥ 2 - 3 | 32.3 (10) | 26.7 (4) |  |
| ≥ 3 | 19.4 (6) | 53.3 (8) |  |
| Education level (years) ^£^ |  |  | 0.372 |
| < 9 | 58.1 (18) | 42.8 (6) |  |
| ≥ 9 - 12 | 22.6 (7) | 28.6 (4) |  |
| ≥ 12 | 19.3 (6) | 28.6 (4) |  |
| Staging ^£^ |  |  | 0.517 |
| I | 22.6 (7) | 20.0 (3) |  |
| II | 25.8 (8) | 6.7 (1) |  |
| III | 25.8 (8) | 40.0 (6) |  |
| IV | 3.2 (1) | 13.3 (2) |  |
| Pathological anatomy Y | 16.1 (5) | 13.3 (2) |  |
| Treatment |  |  | **0.032** |
| Surgery | 9.7 (3) | 20.0 (3) |  |
| Chemotherapy or radiation therapy | 22.6 (7) | 53.3 (8) |  |
| Chemoradiotherapy | 67.7 (21) | 26.7 (4) |  |

* Monthly minimum wage at the time were equivalent to R$998.00.

**^**^**Of the total of 46 participants at the baseline: T0-T1-T2 (n=14), plus T0-T2 (n=23) minus the follow-up losses (5 death + 1 Removed colostomy) equal 31 participants at least two times. So, 15 represents follow-up losses.

^£^ n may vary according to the variability of the data.

* Student's t test (t test) for quantitative and chi-square variables for qualitative variables.
